# “Getting sicker quicker”: Does living in a more deprived neighbourhood mean your health deteriorates faster?

**DOI:** 10.1016/j.healthplace.2011.08.005

**Published:** 2012-03

**Authors:** Anne Ellaway, Michaela Benzeval, Michael Green, Alastair Leyland, Sally Macintyre

**Affiliations:** MRC Social & Public Health Sciences Unit, 4 Lilybank Gardens, Glasgow G12 8RZ, UK

**Keywords:** Area deprivation, Neighbourhood, Self-reported health, Longitudinal study, Health trajectories

## Abstract

Data from the longitudinal West of Scotland Twenty-07 Study: Health in the Community was used to examine whether, over a 20 year period, the self-reported health of people living in deprived areas became poorer faster compared to those living in more affluent areas. Three cohorts (born in the early 1930s, 1950s and 1970s) are included, covering 60 years of the life span. Using multilevel growth curve models, a 40% probability of reporting poor health was predicted among residents of more deprived areas at an earlier age (66) compared to those living in more affluent areas (83). Wider area differences were seen for men than for women. Our findings indicate that attempts to reduce area differences in health should start young but also continue throughout the lifespan.

## Highlights

► Longitudinal data used to examine changes in self-reported health over a 20 year period. ► Health of people in poorer areas deteriorated faster than those in more affluent areas. ► 16 year gap between richer and poorer areas in odds of reporting poor health. ► Wider area differences were seen for men than for women.

## 1 Introduction

A number of studies have shown that living in a more deprived area (variously defined) is associated with poorer health, controlling for individual characteristics such as age, sex and socio-economic status (Pickett and Pearl, 2001; Riva et al., 2007). This has been found for total and coronary heart disease (CHD) mortality (DiezRoux et al., 1997), CHD prevalence and risk factors (Davey Smith et al., 1998) morbidity (Jones and Duncan, 1995; White et al., 2011) mental health and functioning (Beard et al., 2009; Kim 2008; Walters et al., 2004), and health behaviours such as diet, physical activity, smoking and alcohol consumption (Amuzu et al., 2009; Ecob and Macintyre, 2000; Ellaway and Macintyre, 1996). However, most studies to date have been of cross sectional design, which are unable to rule out self-selection (the possibility that people will be selected or select themselves into residential areas on the basis of their health or individual characteristics, which are themselves related to health (Plantinga and Bernell, 2007b, Plantinga and Bernell, 2007a)) and correspondingly limited capacity to explore the plausible causal pathways through which area level exposures might influence health (Macintyre et al., 2002) . Moreover, of the limited number of longitudinal studies, which have been undertaken, most have examined individual and area level exposures at one point in time in relation to health or behavioural outcomes by a single point in time several years later (Giskes et al., 2006; Haan et al., 1987; Pollack et al., 2005, Stafford et al., 2008; Yen and Kaplan, 1998,1999); exceptions include studies of trajectories in Body Mass Index (Ruel et al., 2010; Stafford et al., 2010)). Examining trajectories in health is important for improving our understanding of life course influences on health, as is determining at what age neighbourhood differences are more or less observable (Ahluwalia et al., 2007).

A number of mechanisms through which area of residence may influence health (including major causes of death such as cardiovascular disease (Chaix, 2009; Daniel et al., 2008)), over and above individual characteristics have been suggested. Mechanisms include the hypothesis of differential ‘weathering’ (Bird et al., 2010; Geronimus et al., 2006), which suggests that earlier health deterioration is a consequence of the cumulative impact of repeated experience of social or economic adversity and political marginalisation, and is similar to the ‘more miles on the clock’ metaphor used to explain the earlier health disadvantage experienced by residents of Glasgow compared to residents of Edinburgh, Scotland (Watt and Ecob, 1992; Watt, 1993).

The aim of this paper is to investigate whether the self-reported health of people living in deprived areas becomes poorer over time faster than among residents living in more affluent areas, and to estimate at what age any differences in self-reported health emerge. We also explore whether there are any gender differences in trajectories or age effects, since some studies have found differences between men and women in the magnitude of associations between individual health and experiences of neighbourhood conditions (Chuang and Chuang, 2008; Ellaway and Macintyre, 2001,2009; Kavanagh et al., 2006, Molinari, 1998; Naimi et al., 2009; Parkes and Kearns, 2006; Poortinga et al., 2008; Stafford et al., 2005). To examine these questions we draw upon the ‘West of Scotland Twenty-07 Study’ (Benzeval et al., 2008), which is well placed to address this issue, being sampled from a well-characterised geographic region and using three age cohorts, who have been followed for 20 years thereby encompassing 60 years of the lifespan.

## 2 Data

The Twenty-07 Study has been following people in three age cohorts—born in the early 1930s, 1950s, and 1970s—for 20 years, sampled from the Central Clydeside Conurbation, West of Scotland (Benzeval et al., 2008). Baseline interviews (*n*=4510) were carried out in 1987/1988 when respondents were aged approximately 15, 35 and 55, and there have been four follow-ups (1990/1992; 1995/1997; 2000/2004; 2007/2008). At the most recent wave respondents were aged approximately 35, 55 and 75. Respondents who participated at baseline have been shown to be representative of the general population of the sampled area (Der, 1998). Ethics approval was gained for each wave from appropriate NHS and/or University of Glasgow Research Ethics Committees.

## 3 Measures

The self-assessed health question asked was: *over the last 12 months how would you say that your health on the whole has been…*? *excellent, good, fair, poor.* This has been modelled as a binary outcome (0=‘excellent/good’, 1=‘fair/poor’; with ‘fair/poor’ health hereafter referred to as poor health). Self-assessed health has been shown to be related to specific and all cause mortality (Idler and Benyamini, 1997; Burström and Fredlund, 2001). Area deprivation at baseline is based on Carstairs' score for postcode sectors (average population=5,000) derived from the 1991 Census (McLoone and Boddy, 1994). Carstairs' scores provide an index of deprivation based on an unweighted combination of four census variables comprising the proportion of: households in the area that are overcrowded; heads of household in the area that are in social classes IV and V; male heads of household in the area that are unemployed and households in the area that do not have access to a car. Based on these scores, the areas have been divided into seven deprivation categories (hereafter referred to as depcats); in this paper we have grouped them further for ease of presentation into three groups: 1 and 2, ‘most affluent’; 3, 4 and 5, ‘middling’; 6 and 7, ‘most deprived’. Baseline social class was coded according to the Registrar General’s 1980 classification (OPCS, 1980) for head of household's current or previous occupation. Length of residence at baseline was measured by asking respondents how long they had lived at their current address.

The distribution of respondents at each wave according to these key baseline characteristics is shown in Table 1. The proportion of respondents who were in the oldest cohort, in poor health, in manual classes or living in the most disadvantaged areas at baseline has declined over the 20 years of the study. However, the final column shows the data used for the models in this paper (person-waves) and these are relatively similar to the baseline sample for most characteristics except area deprivation.

## 4 Modelling strategy

Given the clustered nature of the data — both geographically and within individuals — hierarchical repeat measures models, also known as growth curve models, were employed, which use pseudo-maximum likelihood estimators to adjust for non-response under the assumption that any missing data are missing at random (Clarke and Hardy, 2007). Data were included in the analysis for each wave in which respondents participated, thus only missed waves were excluded, reducing potential attrition bias. Multilevel models also allow variation between the original sampling areas to be distinguished from variation between individuals within areas so that the area variation can be examined explicitly. Models were fitted in MLwiN version 2.15 (Rasbash et al., 2009) with three levels-measurement points (level 1, *N*=11,607) nested within individuals (level 2, *N*=3,683) nested within the original sampling units (level 3, *N*=62 postcode sectors). The significance of individual variables was assessed using the Wald test. Baseline social class was split into a dichotomous variable (non-manual vs. manual). To keep the estimates for other parameters neutral (Sacker et al., 2005), gender has been coded −0.5 for men and 0.5 for women, and a similar centring was used for the dichotomised baseline social class variable. Length of residence at baseline and age were included in models as continuous variables (centred on their respective means). Dummy variables for cohort (reference cohort—1950s) were used in most analyses and these were switched to dummy variables for study wave (reference wave—1990/1992) in a range of sensitivity analyses as described below.

The first step was to examine health outcomes at waves 2–5 with explanatory variables (age, gender and social class) measured at wave 1. Modelling was performed in four main stages. First, drawing on other work (Benzeval et al., submitted for publication) the best fitting age function (cubic) was used to model the age–health trajectory, adjusting for gender and cohort, to establish the extent to which self-reported health varied across areas (in this case postcode sectors) and act as a benchmark to compare other findings (Model 1, not shown). Next, to investigate whether area deprivation explained variation across places and whether this varied as respondents aged, area deprivation was added to Model 1 as a main effect and interaction with age (Model 2). Thirdly, we investigated whether any differences by area deprivation could be explained by individual level socio-economic status by adding individuals' social class at baseline (as a main effect and interactions with age) to the model (Model 3). Finally, to understand the extent to which individual effects by themselves explained the between place variation in health trajectories, we also constructed a model using only age, cohort, baseline class and gender without area deprivation (Model 4), and examined the proportion of the area variance explained for each model. Each of these models was repeated stratified by gender. To investigate further the extent to which individual or area deprivation explains the between place differences in the health trajectories, we examined the proportion of area variance in self-rated health explained by the different sets of explanatory factors. The proportion of the total variation that was attributable to areas was calculated following the calculations of intraclass correlation coefficients for logistic regression models based on the assumption of a threshold model (Snijders and Bosker, 1999).

In order to assess whether the associations were robust to adjustment we undertook a range of separate sensitivity analyses. Length of residence at baseline was added to the model and interactions with depcat and depcat by age were tested to see if effects were stronger for those who had been resident in their respective areas for longer periods. The dummy variables for cohort were replaced with dummy variables for study wave to assess period effects. The analysis was also repeated for two sub-samples of respondents: those person-waves where the respondent remained resident in their baseline postcode sector (*n*=7076 person-waves) to remove potential migration effects, and those respondents who had participated in all survey waves (*n*=7368 person-waves) to investigate whether the associations differed for those followed for the full 20 years. Random intercept models were used, with the relationship between age and self-reported health assumed to be constant across areas.

Results are presented graphically as growth curves of predicted probabilities (from the fixed part of the models, the method of constructing these is given in the online supplementary material associated with this paper), with their 95% confidence intervals (represented by the shaded grey bands). The age at which respondents from different area deprivation categories experience the same probability of reporting poor health has also been calculated to illustrate the degree to which those in more disadvantaged areas ‘get sicker’ at younger ages than others.

## 5 Results

**Fig. 1:**
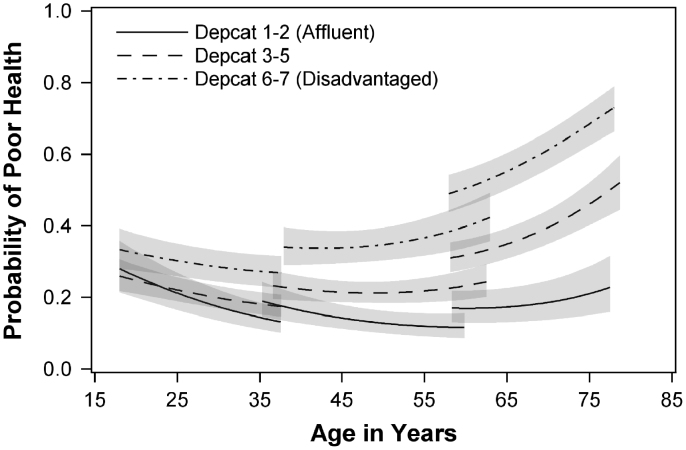
Predicted trajectories in probability of poor health^⁎^ by age and area deprivation, adjusting for gender. (^⁎^Modelling began by examining health at Wave 2–5. There is a slight overlap in ages between consecutive cohorts at Waves 2 and 5 for a small number of cases (due to variations in dates of birth and interview dates)).

Fig. 1 illustrates the poor health trajectories of the three cohorts for the three area deprivation groups, and is derived from Model 2 (i.e. the model with area deprivation, age, cohort, gender and the interaction between area deprivation and age). Respondents living in the most deprived areas at baseline have higher probabilities of reporting poor health than others at all ages; but the gap between them and those living in the most affluent areas widens as people age. The same is true of the middle group (depcats 3, 4 and 5), which has a similar shaped age trajectory, but a lower probability of poor health than that of those from the most deprived areas.

**Fig. 2:**
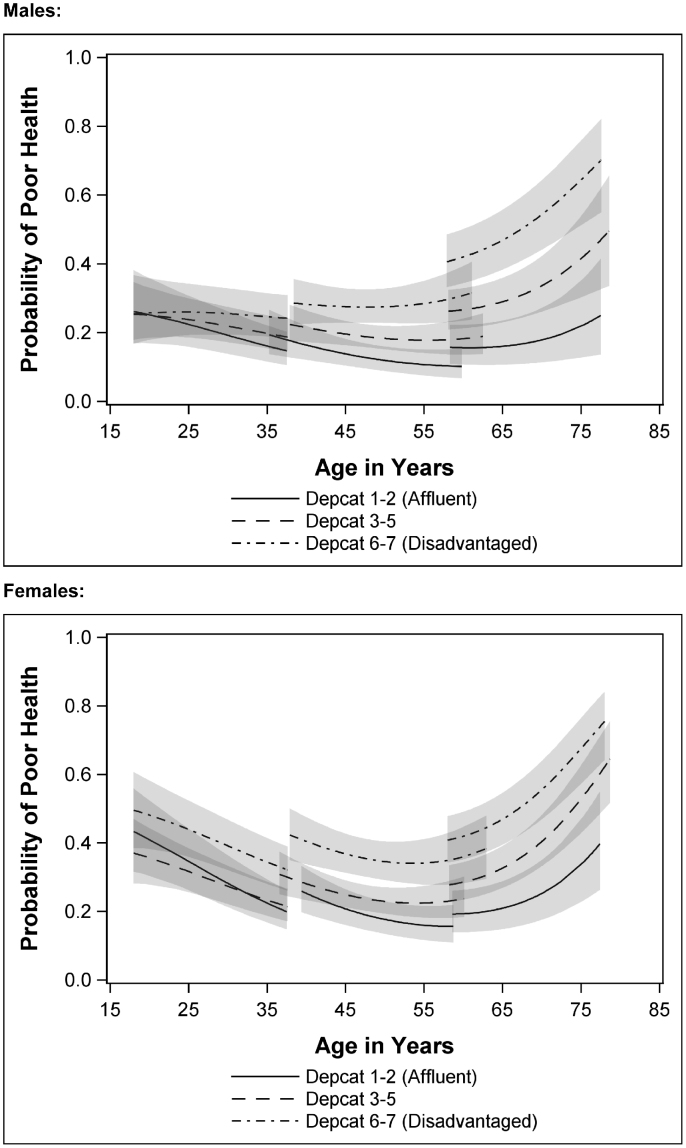
Predicted trajectories in probability of poor health^⁎^ by gender and area deprivation, adjusting for individual social class. (^⁎^Modelling health began with examining health at Waves 2–5, explanatory variables (age, gender, social class and area of residence) are measured at Wave 1).

Fig. 2 shows age-trajectories by area deprivation categories for men and women separately, controlling for individual social class (Model 3). Women have a higher starting level of poor health (at age 18) than men, and while not statistically significant (at the 95% level) more of a difference in *initial* health by area deprivation can be seen for women than for men in the younger cohorts. Inequalities between those living in deprived areas at baseline and others grow for both men and women thereafter, becoming significant around the age of 38. From these ages the area inequalities gap between those living in affluent and deprived areas grows as people age and appears to widen more steeply for men than for women.

In order to investigate further the extent to which individual or area deprivation explains the between place differences in the health trajectories, we examined the proportion of area variance in self-rated health explained by the different sets of explanatory factors. The area level variance accounted for 6.67% of the total variance in the model based on the combined sample of men and women, 5.78% in the male only model and 5.58% in the women only model. Table 2 shows that adding area deprivation (in Model 2) explained almost three quarters of the area level variance (72.3%). The addition of individual social class (Model 3) explained a further 12.3%, suggesting that this factor is also patterned by area of residence. Subtracting the total contribution of class, age, cohort and gender (from Model 4: 44.1%) from the combined effect of area and individual factors, suggests that 40.5% of the area level variation is uniquely explained by area deprivation and its interaction with age. Area deprivation explains relatively less of the area level variation in women than it does in men (Model 2: 93.1% for men compared to 73.9% for women), and for men the combination of area deprivation, class and gender entirely explains the area level variation in health, whereas only 84.7% is explained for women (Model 3). Subtracting the explained variance for individual factors only (Model 4) from these totals suggests that area deprivation uniquely accounts for 40.3% of the area variation in men and 37.9% in women.

Table 3 illustrates the age gap between living in the two most deprived depcats at baseline and elsewhere for different probabilities of reporting ill health. The age estimates in Table 3 are derived from Model 3; they are adjusted for gender and baseline social class. So, for example, those living in the most deprived areas (depcats 6 and 7) at baseline would reach a 40% probability of reporting poor health at around age 66, while those living in the most affluent areas would not reach this probability until they were almost 83 years old, a difference of around 16 years. Even those living in the ‘middling’ deprived areas at baseline will not reach the same probability of ill health until 9 years after those in the poorest areas, at the age of 75. For men the difference between the most and least deprived areas was almost 20 years, while for women the same difference was just over 15 years, showing wider area inequalities in men’s compared to women's health.

A number of sensitivity analyses were performed (results are provided in the online supplementary material associated with this paper). The relationship between area deprivation, age and the probability of poor health was found to be robust to adjustment for period effects and the length of residence at the baseline postcode sector. Repeating the modelling using only those person-waves where respondents were still resident at their baseline postcode at the end of the 20-year follow-up did not substantively alter the results, nor did repeating the modelling using only those respondents who had participated at every wave.

## 6 Discussion

We have shown that, during a 20 year period between 1987 and 2007, in a large urban and periurban region in the West of Scotland, there are differences in reporting poor self-rated health by deprivation of place of residence at baseline, controlling for age, cohort, gender and socioeconomic status; with those in poorer neighbourhoods reporting poor health at much younger ages, and the likelihood of reporting poor health in deprived neighbourhoods increasing more steeply with age, than for those in better off neighbourhoods. Adding to current debates about differential place effects by gender (Frye et al., 2008), we also found wider area differences in old-age for men than for women, although overall, women were more likely to report poor health at an earlier age than men. These findings were robust for control for period effects and length of residence. The estimated age differences in the probability of reporting poor health are substantial; for a 40% probability, the middling areas would report poor health some 9 years after those in the poorest neighbourhoods, and those in the richest neighbourhoods would not have this probability of reporting poor health for an additional 7 years.

There are a number of important implications of these findings, some methodological, some theoretical and some practical. Our analysis of area differences reveals important evidence as to how the relationship between age and poor health not only varies by deprivation categories but also how age-*trajectories* vary by place of residence. Routinely controlling for age in an analysis of area differences may well mask such evidence. One theoretical implication is that both low socioeconomic status and neighbourhood deprivation seem to have cumulative, long-term, effects on self-reported health. Whether this results from a critical period process (living in poor circumstances at a particular stage in life programmes the body to deteriorate more rapidly subsequently) or from cumulative exposure or vulnerability to adverse, and possibly worsening, circumstances cannot be answered from this analysis, but could be the subject of further analysis on this or other cohorts.

Practical implications are that neighbourhood deprivation differences in poor self rated health may start relatively early in life, and exist over and above individual socio-economic differences. This suggests that attempts to reduce inequalities in health should focus on places as well as people, and recognise a greater burden of morbidity among both poor places and poor people. The other major practical implication is that attempts to reduce area inequalities in health should start young (Curtis et al., 2004), to avoid the initial development of area differences, but also may need to be continued throughout the lifespan to avoid continued divergence between social groups.

### 6.1 Limitations

While the Twenty-07 Study ranges in age from 15 to 76 (in three separate cohorts), it does not cover childhood or older old-age. This is a limitation shared in much of the literature, reflecting the length and scope of many longitudinal studies. This paper focuses on self-assessed health, which has been shown to be a good predictor of mortality and morbidity, but the way people answer the question may change with age and period (Hoeymans et al., 1997). It has been hypothesised that there may also be SES differences in response to this question (Burström and Fredlund, 2001; Singh-Manoux et al., 2007). However, we found this was not the case with longstanding illness in the Twenty-07 Study (Macintyre et al., 2005). In this analysis we have looked at area of residence at baseline, rather than at every wave of contact, so have not been able to examine moves between different types of area, or any self-selective processes involved in residential migration. Similarly, and by definition, we have not been able to examine health or other social characteristics preceding the residence ascertained at baseline, so have not been able to examine selection processes directly. Finally, our measure of the area of residence is based on postal geography, not respondent defined neighbourhoods, and a pre-existing measure of area deprivation, rather than a richer measure of area characteristics based on a larger set of contextual measures. However the Carstairs score was developed in a way which captured postcode sector differences in mortality, and is widely used in health geography in the UK.

Strengths of this study are that it uses a general population sample from a socially and geographically heterogeneous region, rather than an occupational cohort (e.g. Stafford et al., 2008), covers 60 years of the life course, can control for period and cohort effects, and includes a range of small areas with varying social and material conditions.

Our findings are consistent with those reported from other studies in a range of different countries in showing an association between area deprivation and poor self-rated health, controlling for individual characteristics (Pickett and Pearl, 2001; Riva et al., 2007). However we have been able to add to these predominantly cross-sectional studies by showing that this association strengthens with age, and that these trajectories are robust to control for prior length of residence and for remaining in the area. These results are consistent with the hypothesis of differential ‘weathering’ (Bird et al., 2010; Geronimus et al., 2006) and our findings suggest that this weathering process is related not only to individual or family socioeconomic adversity, but also to area level adversity.

## Figures and Tables

**Table 1:** Proportion (%) of respondents taking part in each wave by their baseline characteristics.

Baseline characteristics	Wave 1 1987/1988	Wave 2 1990/1992	Wave 3 1995/1997	Wave 4 2000/2004	Wave 5 2007/2008	Modelling data^a^
% 1970s cohort	33.6	35.2	30.8	31.7	36.2	34.1
% 1950s cohort	32.0	31.9	34.5	36.8	38.4	33.3
% 1930s cohort	34.4	33.0	34.7	31.5	25.5	32.6

% men	46.5	46.1	44.6	45.0	44.6	46.2
% women	53.5	53.9	55.4	55.0	55.4	53.8

% good starting health^b^	72.7	76.1	76.6	78.6	80.4	78.1
% poor starting health^b^	23.4	22.8	22.3	20.3	18.2	20.8
% missing starting health^b^	3.9	1.1	1.1	1.1	1.4	1.1

% non-manual classes	42.0	43.7	46.3	48.0	48.5	48.0
% manual classes	54.0	52.9	50.6	48.6	47.6	52.0
% missing class	4.0	3.5	3.1	3.5	3.9	N/A

% depcat 1 or 2 (affluent)	15.2	15.9	16.9	17.9	18.4	17.5
% depcat 3, 4 or 5	45.9	47.4	49.6	49.0	48.6	49.0
% depcat 6 or 7 (disadvantaged)	38.8	36.6	33.5	33.1	33.0	33.5
Total interviewed	4510	3820	2972	2661	2604	11607

a Data modelled as person-waves using Waves 2–5.

b Question wording and response categories for self-rated health differed slightly for the 1970s cohort at baseline.

**Table 2:** Proportion of variance explained by each model.

Model	Variables	Percentage of model 1 area level variance explained
All		
1	Constant, age, age-squared, age-cubed, gender and cohort	n/a
2	As model 1 adding depcat, and depcat×age^a^	72.2
3	As model 2 adding class, class×age, class×age-squared, class×age-cubed	84.6
4	As model 3 without depcat or depcat×age	44.1

Males		
1	Constant, age, age-squared, age-cubed, and cohort	n/a
2	As model 1 adding depcat, and depcat×age^a^	93.1
3	As model 2 adding class, class×age, class×age-squared, class×age-cubed	100.0
4	As model 3 without depcat or depcat×age	59.7

Females		
1	Constant, age, age-squared, age-cubed, and cohort	n/a
2	As model 1 adding depcat, and depcat×age^a^	73.9
3	As model 2 adding class, class×age, class×age-squared, class×age-cubed	84.7
4	As model 3 without depcat or depcat×age	46.8

a Interactions between depcat and age-squared or age-cubed were left out as they were not significant at the *p*<0.05 level in most models. For females only the interaction effect between age-squared and being in the most deprived category was significant in Model 2 but not in model 3, suggesting that this was mainly due to effects of individual class, and so this has been left out for consistency with the overall and male only models.

**Table 3:** Ages at which particular probabilities for reporting poor health are predicted for respondents in different area deprivation categories.

*Age at which probability of poor health is attained*…	Age^a^ (difference^b^) all			Age^a^ (difference^b^) males			Age^a^ (difference^b^) females		
	Depcats 6 and 7	Depcat 3–5	Depcat 1 and 2	Depcat 6 and 7	Depcat 3–5	Depcat 1 and 2	Depcat 6 and 7	Depcat 3–5	Depcat 1 and 2
Probability of 0.4^c^	66.4 (−)	75.4 (+9.0)	82.8 (+16.4)	68.6 (−)	79.5 (+10.9)	88.1 (+19.5)	64.2 (−)	72.6 (+8.4)	79.4 (+15.2)
Probability of 0.5^c^	71.9 (−)	78.9 (+7.0)	85.5 (+13.6)	73.9 (−)	83.0 (+9.1)	90.7 (+16.8)	70.3 (−)	76.2 (+5.9)	82.2 (+11.9)
Probability of 0.6^c^	75.8 (−)	81.8 (+6.0)	87.8 (+12.0)	77.9 (−)	85.9 (+8.0)	93.0 (+15.1)	74.4 (−)	79.1 (+4.7)	84.7 (+10.3)

a Values for all respondents are adjusted for gender and baseline social class, and figures for males and females are adjusted for baseline social class (i.e. Model 3). All values are calculated for a respondent from the 1950s cohort.

b The term in brackets represents the predicted difference in years with depcats 6 and 7 as the reference category.

c In some instances the model also predicts a probability of poor health at or above this level at younger ages as well but the focus of comparison was the point at which the predicted probability curve crosses from below to above these thresholds with increasing age.
