# Supplementary material for: “Getting sicker quicker”: Does living in a more deprived neighbourhood mean your health deteriorates faster?
Source: Health Place. 2012 Mar;18-20(2):132–7. doi: 10.1016/j.healthplace.2011.08.005 (PMC3391685; doi:10.1016/j.healthplace.2011.08.005)
Supplement: Supplementary file 1 — Supplementary materials [file mmc1.doc]

**“Getting Sicker Quicker”: does living in a more deprived neighbourhood mean your health deteriorates faster?**

**MS Ref#: JHAP-D-10-00434**

**Supplementary Information**

*Part I: Details of Analysis Models*

Supplementary Tables 1-3 display parameter estimates for models 1-4. Supplementary Table 1 shows those for models with all respondents whilst Supplementary Tables 2 and 3 respectively display estimates for models with males and females only. Estimates which differ significantly from zero are displayed in bold. In order to make the coefficients interpretable within the number of decimal places presented here age^2^ was divided by 100 before inclusion in the models and age^3^ was divided by 10,000. Age is centred on the grand mean of 46.4 years and gender and social class are centred on zero (-0.5=male, 0.5=female; -0.5=non-manual social class, 0.5=manual social class).

**Supplementary Table 1: Details of models for all respondents**

| **N=11,607** | **Model 1** | | **Model 2** | | **Model 3** | | **Model 4** | |
| --- | --- | --- | --- | --- | --- | --- | --- | --- |
|  | Estimate | S.E. | Estimate | S.E. | Estimate | S.E. | Estimate | S.E. |
| *Coefficients:* | | | | | | | | |
| Intercept | **-1.153** | **0.091** | **-1.832** | **0.138** | **-1.648** | **0.129** | **-1.162** | **0.081** |
| Age | -0.005 | 0.007 | **-0.026** | **0.008** | **-0.040** | **0.009** | **-0.025** | **0.008** |
| Age^2^ | **0.079** | **0.011** | **0.081** | **0.011** | **0.105** | **0.016** | **0.106** | **0.016** |
| Age^3^ | 0.072 | 0.061 | 0.072 | 0.062 | **0.268** | **0.086** | **0.260** | **0.085** |
| Gender | **0.207** | **0.073** | **0.207** | **0.073** | **0.230** | **0.073** | **0.235** | **0.073** |
| 1970s Cohort | **-0.350** | **0.138** | **-0.342** | **0.139** | **-0.342** | **0.138** | **-0.341** | **0.138** |
| 1950s Cohort (ref) | **-** | **-** | **-** | **-** | **-** | **-** | **-** | **-** |
| 1930s Cohort | **0.484** | **0.131** | **0.433** | **0.132** | **0.350** | **0.132** | **0.387** | **0.131** |
| Depcat1-2 (ref) | - | - | **-** | **-** | **-** | **-** | - | - |
| Depcat3-5 | - | - | **0.527** | **0.144** | **0.354** | **0.132** | - | - |
| Depcat3-5*Age | - | - | **0.022** | **0.006** | **0.017** | **0.006** | - | - |
| Depcat6-7 | - | - | **1.170** | **0.151** | **0.888** | **0.142** | - | - |
| Depcat6-7*Age | - | - | **0.032** | **0.006** | **0.025** | **0.006** | - | - |
| Class | - | - | - | - | **0.661** | **0.102** | **0.719** | **0.100** |
| Class*Age | - | - | - | - | **0.038** | **0.008** | **0.043** | **0.008** |
| Class*Age^2^ | - | - | - | - | **-0.041** | **0.020** | **-0.043** | **0.020** |
| Class*Age^3^ | - | - | - | - | **-0.388** | **0.109** | **-0.377** | **0.109** |
| *Variation:* | | | | | | | | |
| Area | **0.227** | **0.057** | **0.063** | **0.027** | 0.035 | 0.021 | **0.127** | **0.038** |
| Individual | **2.407** | **0.110** | **2.456** | **0.113** | **2.402** | **0.112** | **2.354** | **0.109** |
| Time-Point | 1.000 | - | 1.000 | - | 1.000 | - | 1.000 | - |

**Supplementary Table 2: Details of models for male respondents**

| **N=5,363** | **Model 1** | | **Model 2** | | **Model 3** | | **Model 4** | |
| --- | --- | --- | --- | --- | --- | --- | --- | --- |
|  | Estimate | S.E. | Estimate | S.E. | Estimate | S.E. | Estimate | S.E. |
| *Coefficients:* | | | | | | | | |
| Intercept | **-1.369** | **0.118** | **-2.066** | **0.169** | **-1.881** | **0.165** | **-1.367** | **0.107** |
| Age | 0.001 | 0.010 | **-0.022** | **0.012** | **-0.036** | **0.013** | -0.020 | 0.012 |
| Age^2^ | **0.076** | **0.017** | **0.076** | **0.017** | **0.074** | **0.025** | **0.074** | **0.025** |
| Age^3^ | 0.009 | 0.092 | 0.009 | 0.093 | 0.231 | 0.132 | 0.223 | 0.132 |
| 1970s Cohort | -0.243 | 0.211 | -0.229 | 0.209 | -0.234 | 0.207 | -0.241 | 0.210 |
| 1950s Cohort (ref) | **-** | **-** | **-** | **-** | **-** | **-** | **-** | **-** |
| 1930s Cohort | **0.699** | **0.193** | **0.621** | **0.191** | **0.486** | **0.193** | **0.533** | **0.195** |
| Depcat1-2 (ref) | - | - | **-** | **-** | **-** | **-** | - | - |
| Depcat3-5 | - | - | **0.592** | **0.170** | **0.442** | **0.165** | - | - |
| Depcat3-5*Age | - | - | **0.020** | **0.008** | 0.017 | 0.009 | - | - |
| Depcat6-7 | - | - | **1.197** | **0.177** | **0.910** | **0.178** | - | - |
| Depcat6-7*Age | - | - | **0.038** | **0.009** | **0.033** | **0.009** | - | - |
| Class | - | - | - | - | **0.504** | **0.151** | **0.623** | **0.150** |
| Class*Age | - | - | - | - | **0.037** | **0.012** | **0.044** | **0.012** |
| Class*Age^2^ | - | - | - | - | 0.007 | 0.030 | 0.008 | 0.030 |
| Class*Age^3^ | - | - | - | - | **-0.420** | **0.167** | **-0.414** | **0.167** |
| *Variation:* | | | | | | | | |
| Area | **0.216** | **0.075** | 0.015 | 0.033 | 0.000 | 0.000 | 0.087 | 0.049 |
| Individual | **2.521** | **0.175** | **2.484** | **0.172** | **2.417** | **0.166** | **2.488** | **0.172** |
| Time-Point | 1.000 | - | 1.000 | - | 1.000 | - | 1.000 | - |

**Supplementary Table 3: Details of models for female respondents**

| **N=6,244** | **Model 1** | | **Model 2** | | **Model 3** | | **Model 4** | |
| --- | --- | --- | --- | --- | --- | --- | --- | --- |
|  | Estimate | S.E. | Estimate | S.E. | Estimate | S.E. | Estimate | S.E. |
| *Coefficients:* | | | | | | | | |
| Intercept | **-0.981** | **0.109** | **-1.603** | **0.167** | **-1.401** | **0.161** | **-0.999** | **0.101** |
| Age | -0.010 | 0.009 | **-0.031** | **0.011** | **-0.043** | **0.012** | **-0.029** | **0.011** |
| Age^2^ | **0.083** | **0.015** | **0.084** | **0.015** | **0.126** | **0.020** | **0.128** | **0.020** |
| Age^3^ | 0.125 | 0.082 | 0.125 | 0.083 | **0.285** | **0.112** | **0.275** | **0.112** |
| 1970s Cohort | **-0.454** | **0.187** | **-0.453** | **0.186** | **-0.449** | **0.185** | **-0.435** | **0.185** |
| 1950s Cohort (ref) | **-** | **-** | **-** | **-** | **-** | **-** | **-** | **-** |
| 1930s Cohort | 0.341 | 0.180 | 0.284 | 0.180 | 0.251 | 0.180 | 0.300 | 0.180 |
| Depcat1-2 (ref) | - | - | **-** | **-** | **-** | **-** | - | - |
| Depcat3-5 | - | - | **0.465** | **0.171** | 0.261 | 0.164 | - | - |
| Depcat3-5*Age | - | - | **0.024** | **0.008** | **0.018** | **0.008** | - | - |
| Depcat6-7 | - | - | **1.130** | **0.178** | **0.827** | **0.175** | - | - |
| Depcat6-7*Age | - | - | **0.028** | **0.008** | **0.020** | **0.008** | - | - |
| Class | - | - | - | - | **0.819** | **0.135** | **0.925** | **0.134** |
| Class*Age | - | - | - | - | **0.036** | **0.011** | **0.039** | **0.010** |
| Class*Age^2^ | - | - | - | - | **-0.079** | **0.026** | **-0.081** | **0.026** |
| Class*Age^3^ | - | - | - | - | **-0.338** | **0.144** | **-0.322** | **0.144** |
| *Variation:* | | | | | | | | |
| Area | **0.203** | **0.065** | 0.053 | 0.036 | 0.031 | 0.031 | **0.108** | **0.047** |
| Individual | **2.437** | **0.150** | **2.431** | **0.150** | **2.346** | **0.147** | **2.365** | **0.148** |
| Time-Point | 1.000 | - | 1.000 | - | 1.000 | - | 1.000 | - |

*Part II: Details of Calculations for Age Thresholds for Particular Probabilities of Poor Health by Deprivation Category*

Logistic regression estimates the probability of an outcome as follows, where e is the base of the natural logarithm, a is a constant and b is a set of beta coefficients for X explanatory variables:


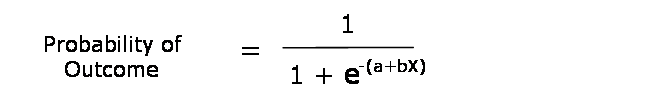


The probability of poor health predicted by the model for people in particular deprivation categories at particular ages can be obtained using the following formulae derived from the logistic regression coefficients in the analysis models. As the model coefficients were calculated with age centred on the grand mean (46.4 years), with age^2^ divided by 100 and with age^3^ divided by 10,000, these transformations should be performed before inclusion in the following equations. In order to obtain gender and social class adjusted estimates, the coefficients from model 3 are used with the coefficients for sex and social class, which were centred on 0, omitted.


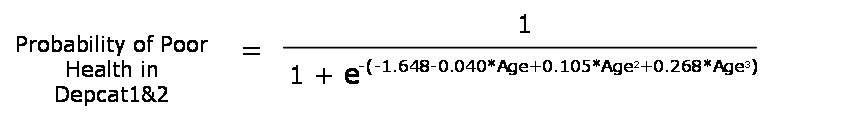


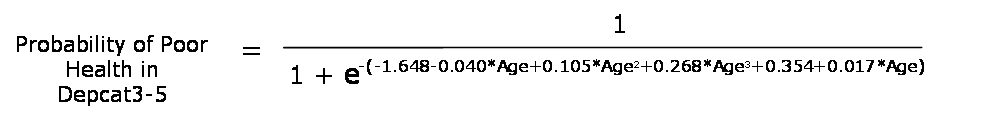


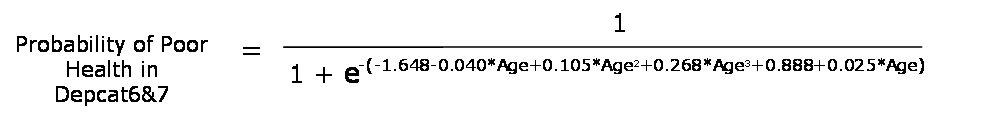


These formulae can then be used to identify the age thresholds at which a certain probability of poor health is reached.

*Part III: Details of Sensitivity Analyses*

Supplementary Table 4 displays parameter estimates for each of the sensitivity analyses conducted. These models are based on model 3 from supplementary Table 1. From left to right: the length of residence (LoR) model includes a further variable for the number of years a respondent had been resident at their baseline postcode sector before the start of the study, and tests whether this interacts with the effects of depcat; the period effects model replaces the dummy variables for cohort with dummy variables for study wave (using the second wave as the reference category); the ‘stayers only’ model includes only those person-waves where a respondent was still resident in their baseline postcode sector (e.g. if a person moved away from their baseline postcode sector between waves three and four then waves four and five would be excluded for that individual, but waves two and three would be included); and finally the complete cases model includes only those respondents who participated in every wave of the study.

**Supplementary Table 4: Details of Sensitivity Analyses**

|  | **LoR**  **(n=10,498)** | | **Period Effects**  **(n=11,607)** | | **Stayers Only (n=7,076)** | | **Complete Cases (n=7,368)** | |
| --- | --- | --- | --- | --- | --- | --- | --- | --- |
|  | Estimate | S.E. | Estimate | S.E. | Estimate | S.E. | Estimate | S.E. |
| *Coefficients:* | | | | | | | | |
| Intercept | **-1.679** | **0.144** | **-1.670** | **0.130** | **-1.695** | **0.163** | **-1.685** | **0.138** |
| Age | **-0.033** | **0.009** | **-0.022** | **0.007** | -0.017 | 0.013 | **-0.041** | **0.011** |
| Age^2^ | **0.104** | **0.017** | **0.116** | **0.015** | **0.117** | **0.024** | **0.129** | **0.020** |
| Age^3^ | **0.235** | **0.091** | **0.253** | **0.086** | 0.189 | 0.126 | **0.322** | **0.104** |
| Female | **0.234** | **0.078** | **0.227** | **0.073** | **0.195** | **0.091** | **0.329** | **0.101** |
| 1970s Cohort | -0.218 | 0.151 | **-** | **-** | -0.015 | 0.255 | **-0.577** | **0.180** |
| 1950s Cohort (ref) | **-** | **-** | **-** | **-** | - | - | - | - |
| 1930s Cohort | **0.350** | **0.141** | **-** | **-** | 0.181 | 0.182 | -0.067 | 0.174 |
| Wave 2: 91-92 (ref) | **-** | **-** | - | - | **-** | **-** | - | - |
| Wave 3: 95-96 | **-** | **-** | 0.113 | 0.067 | **-** | **-** | - | - |
| Wave 4: 00-04 | **-** | **-** | 0.106 | 0.075 | **-** | **-** | - | - |
| Wave 5: 07-08 | **-** | **-** | **-0.304** | **0.088** | **-** | **-** | - | - |
| Depcat1-2 (ref) | **-** | **-** | **-** | **-** | **-** | **-** | - | - |
| Depcat3-5 | **0.386** | **0.149** | **0.356** | **0.132** | **0.341** | **0.167** | 0.156 | 0.140 |
| Depcat3-5*Age | **0.018** | **0.008** | **0.017** | **0.006** | 0.012 | 0.007 | **0.014** | **0.007** |
| Depcat6-7 | **0.888** | **0.160** | **0.893** | **0.142** | **0.887** | **0.180** | **0.732** | **0.157** |
| Depcat6-7*Age | **0.026** | **0.009** | **0.025** | **0.006** | **0.023** | **0.008** | **0.026** | **0.008** |
| Manual Class | **0.723** | **0.109** | **0.673** | **0.102** | **0.884** | **0.142** | **0.623** | **0.132** |
| Manual*Age | **0.032** | **0.009** | **0.039** | **0.008** | **0.029** | **0.011** | **0.038** | **0.010** |
| Manual*Age^2^ | **-0.045** | **0.021** | **-0.044** | **0.020** | **-0.070** | **0.027** | **-0.040** | **0.025** |
| Manual*Age^3^ | **-0.326** | **0.116** | **-0.405** | **0.110** | -0.288 | 0.156 | **-0.379** | **0.138** |
| LoR | -0.008 | 0.015 | **-** | **-** | - | - | **-** | **-** |
| LoR*Depcat3-5 | 0.013 | 0.017 | **-** | **-** | - | - | **-** | **-** |
| LoR*  Depcat3-5*Age | -0.000 | 0.000 | **-** | **-** | - | - | **-** | **-** |
| LoR*Depcat6-7 | -0.002 | 0.018 | **-** | **-** | - | - | **-** | **-** |
| LoR*  Depcat6-7*Age | 0.000 | 0.001 | **-** | **-** | - | - | **-** | **-** |
| *Variation:* | | | | | | | | |
| Area | 0.051 | 0.026 | 0.035 | 0.021 | 0.058 | 0.033 | 0.000 | 0.000 |
| Individual | **2.501** | **0.121** | **2.412** | **0.111** | **2.632** | **0.151** | **2.384** | **0.146** |
| Time-Point | 1.000 | - | 1.000 | - | 1.000 | - | 1.000 | - |
